# Supplementary figures and images for: Molecular docking, simulation and binding free energy analysis of small molecules as PfHT1 inhibitors
Source: PLoS One. 2022 Aug 26;17(8):e0268269. doi: 10.1371/journal.pone.0268269 (PMC9417013; doi:10.1371/journal.pone.0268269)

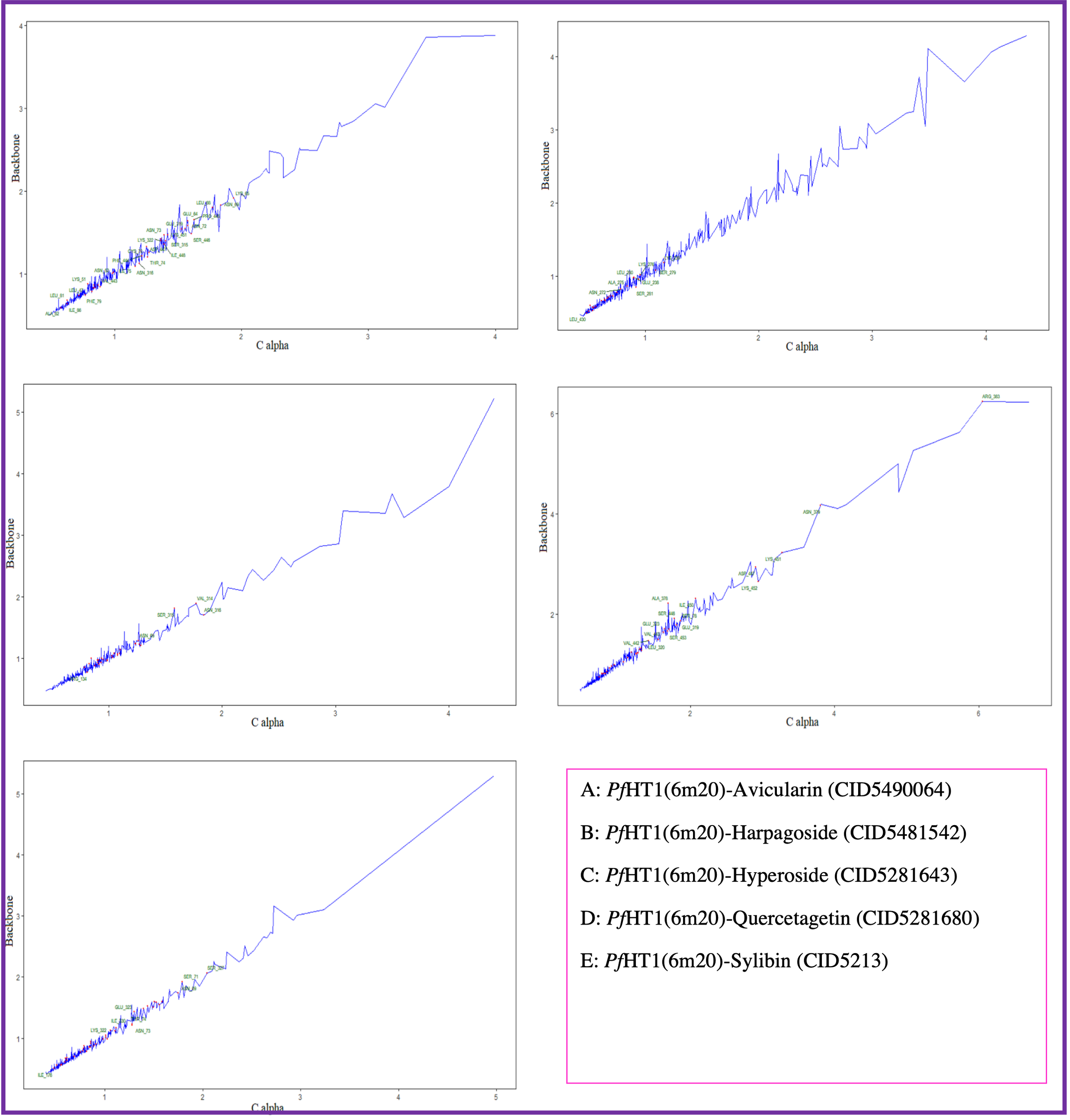

Supplement: S1 Fig — (TIF) [file pone.0268269.s001.tif]

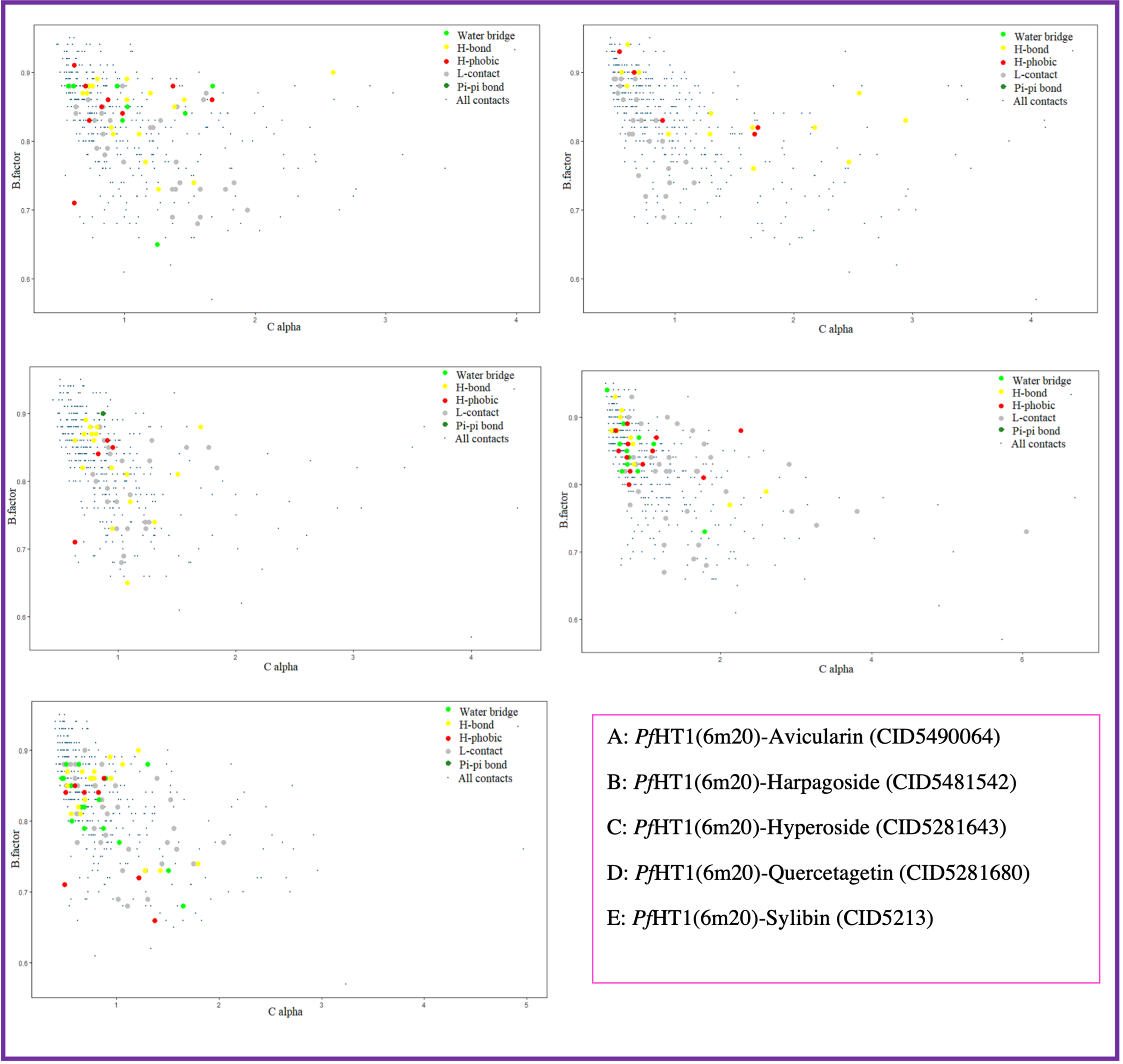

Supplement: S2 Fig — (TIF) [file pone.0268269.s002.tif]

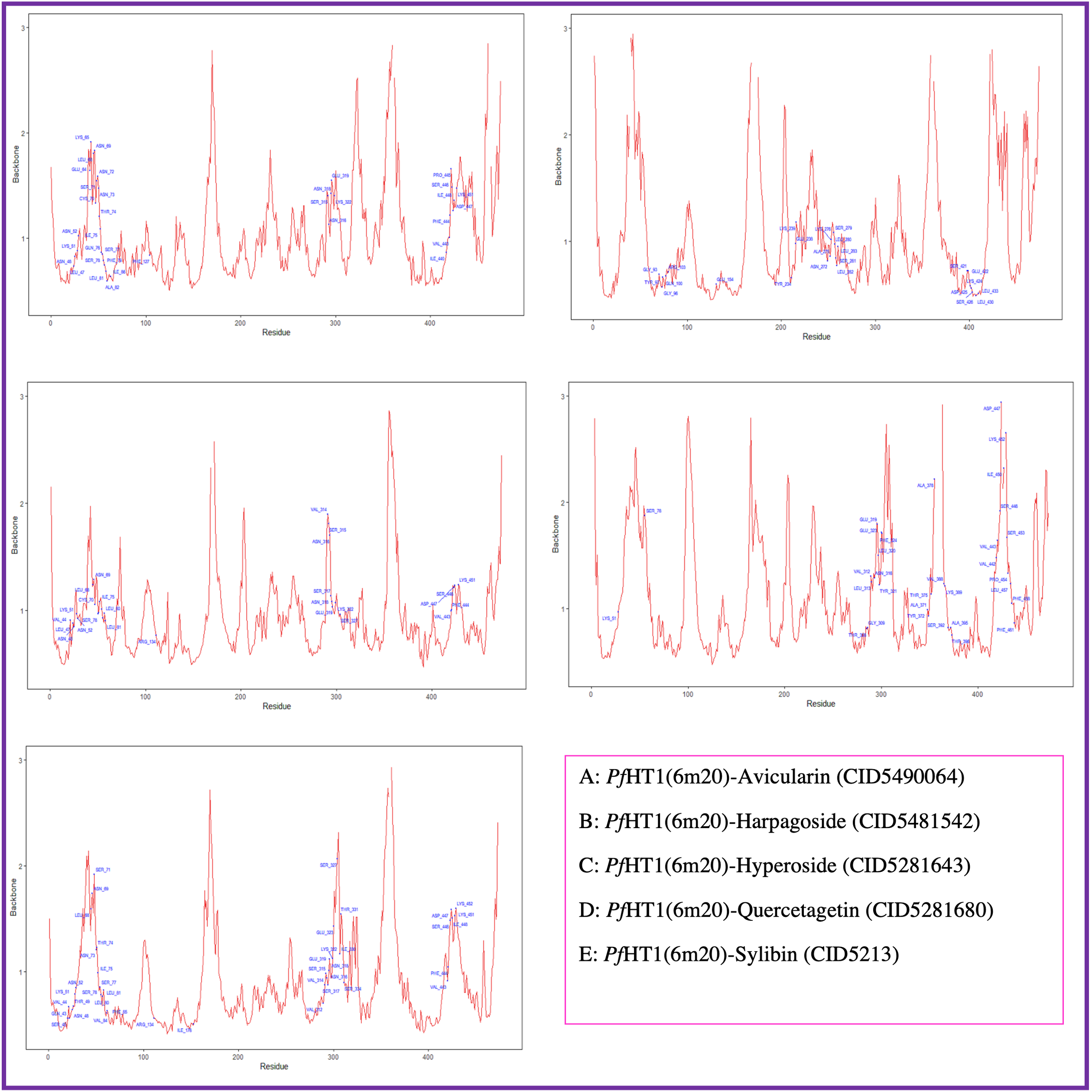

Supplement: S3 Fig — (TIF) [file pone.0268269.s003.tif]

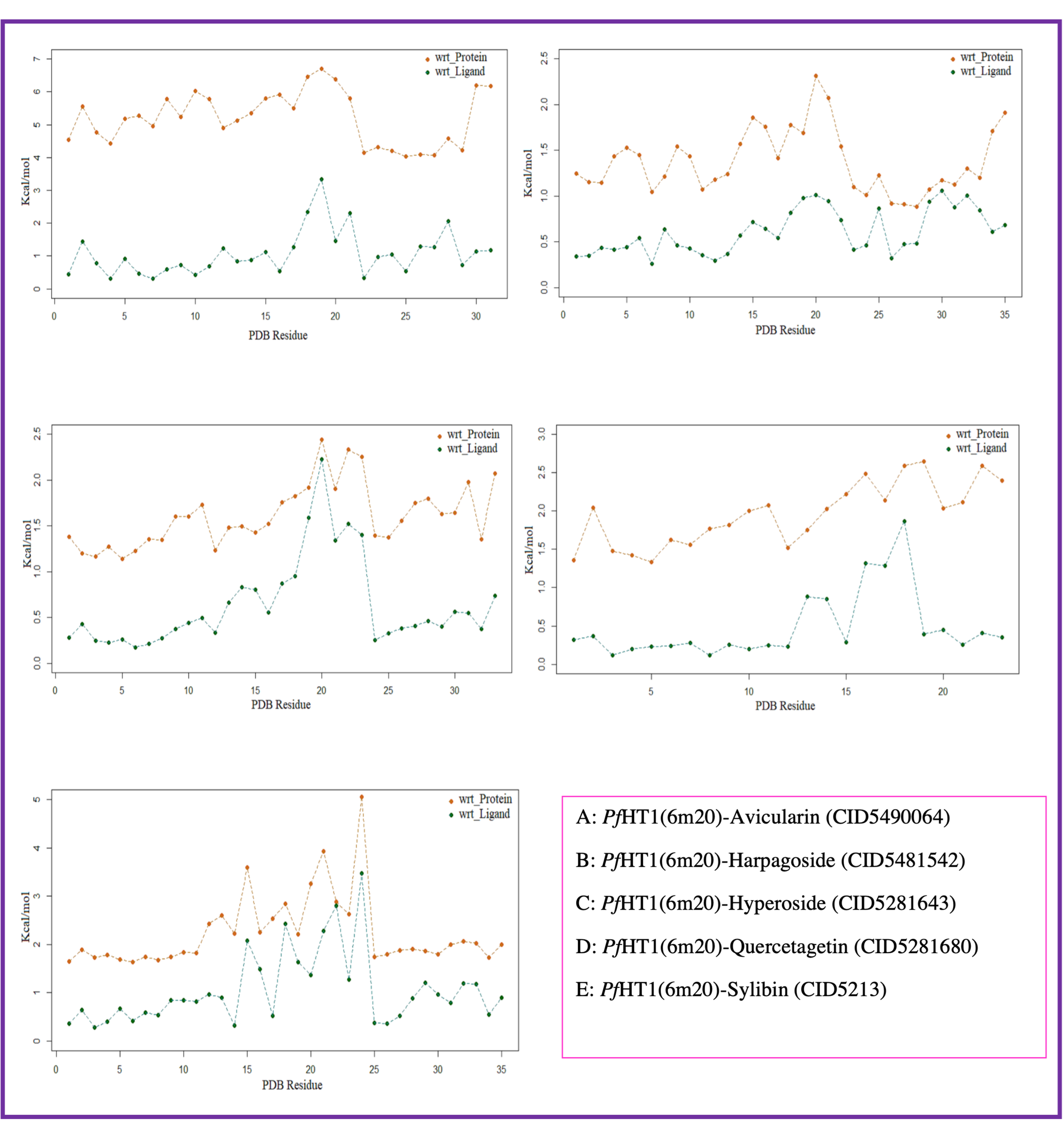

Supplement: S4 Fig — (TIF) [file pone.0268269.s004.tif]

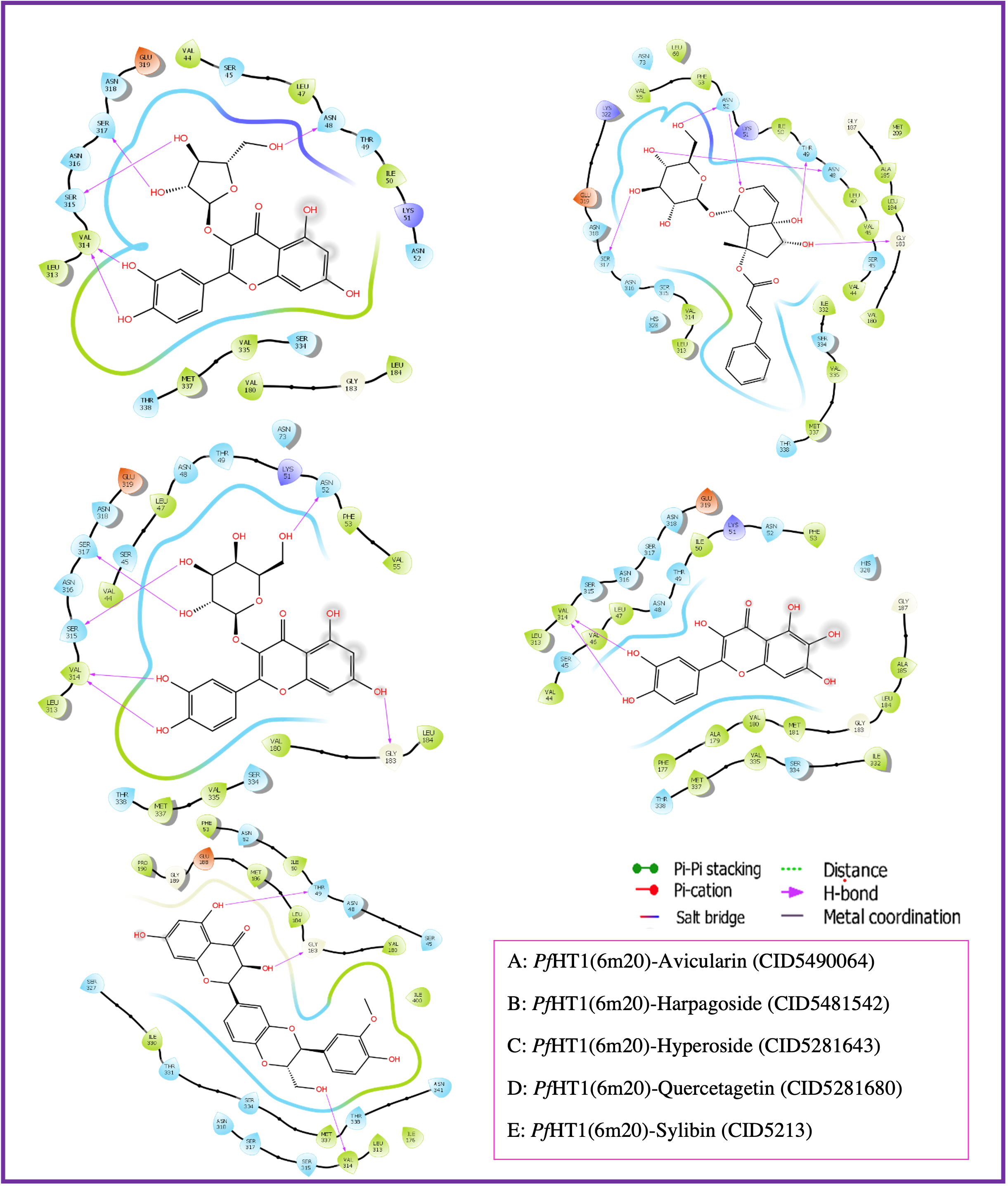

Supplement: S5 Fig — (TIF) [file pone.0268269.s005.tif]

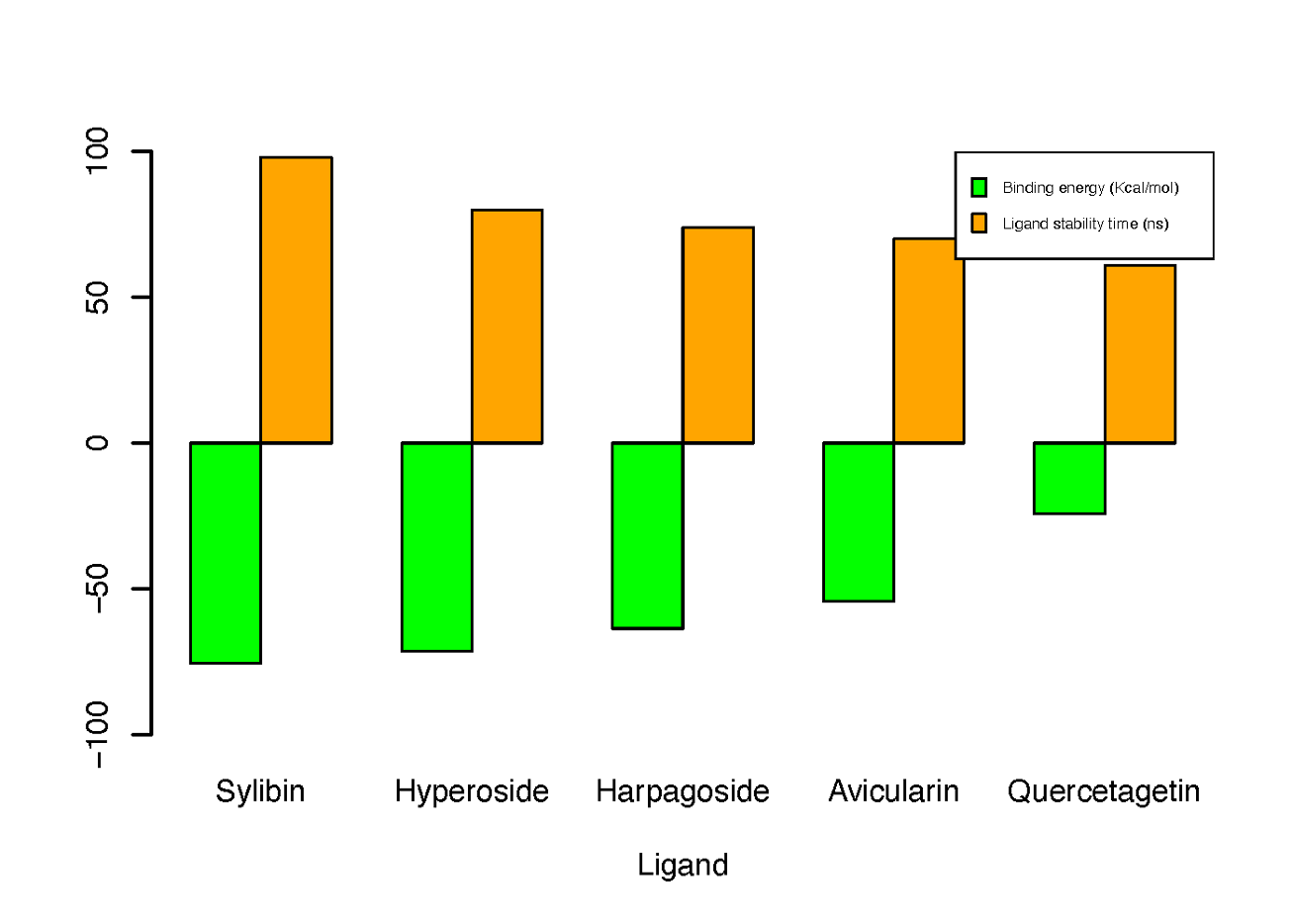

Supplement: S6 Fig — (TIF) [file pone.0268269.s006.tif]
